# Nonessential amino acid is not nonessential in geriatric patients: implications for maxillofacial wound healing and bone repair

**DOI:** 10.1186/s40902-025-00465-w

**Published:** 2025-05-26

**Authors:** Seong-Gon Kim

**Affiliations:** Gangneung–Wonju National University, Gangneung, Republic of Korea

**Keywords:** Nonessential amino acid, Osteoporosis, Wound healing, Bone regeneration, Silk protein

## Abstract

**Background:**

Nonessential amino acids (NEAAs) are traditionally regarded as dispensable because they can be synthesized endogenously from glucose-derived intermediates. Emerging evidence, however, shows that the capacity for de novo NEAA biosynthesis declines in aged tissues, rendering several of these molecules conditionally essential during periods of stress such as surgery or fracture repair.

**Main body:**

In the cranio-maxillofacial arena — where bone and soft-tissue regeneration must occur in an environment already compromised by osteoporosis, multimorbidity, and restricted oral intake — insufficient NEAA supply may translate into delayed union, wound dehiscence, and heightened infection risk. This narrative review integrates biochemical, preclinical, and clinical data to map age-dependent changes in the serine/glycine, glutamine/glutamate, arginine/citrulline, cysteine/trans-sulfuration, and alanine cycles, examines their impact on osteogenesis and mucosal healing, and evaluates nutritional or pharmacological strategies to restore NEAA sufficiency. Particular attention is paid to serine-one-carbon metabolism, the intestinal-renal arginine axis, and redox-sensitive cysteine pathways, all of which are intimately linked to collagen deposition, osteoblast differentiation, and immune modulation.

**Conclusion:**

We conclude that proactive optimization of NEAA status — through targeted supplementation or metabolic activation — represents a low-risk, biologically rational adjunct to enhance postoperative outcomes in geriatric maxillofacial patients.

## Background

The canonical division of amino acids into essential and nonessential categories rests on the premise that the latter can be synthesized endogenously in quantities sufficient for physiological needs. However, the enzymatic network that channels glycolytic and tricarboxylic acid (TCA) cycle intermediates into serine, glycine, glutamate, and related amino acids undergoes tissue-specific remodeling with age — declining in muscle, bone, and the liver yet showing compensatory upregulation in certain neural and glial populations [1, 2]. Transcriptomic surveys of human and murine skeletal muscle, liver, and endothelial tissue demonstrate downregulation of phosphoglycerate dehydrogenase (PHGDH) [3] and phosphoserine aminotransferase (PSAT1) [4] after midlife, paralleled by a fall in circulating serine concentrations [1]. Although plasma arginine can fall in some older individuals, most studies indicate that renal argininosuccinate synthetase-1 (ASS1) expression is maintained with age, and intestinal ASS1 is already low by early adulthood [5]. Thus, age-related impairment of the intestinal-renal arginine axis is more likely driven by reduced renal perfusion and altered arginine utilization than by a late-life decline in ASS1 itself [6]. Collectively, these molecular changes create functional bottlenecks whenever anabolic demand spikes — during infection, fracture, or surgery — rendering traditionally “nonessential” amino acids (NEAAs) conditionally essential in older adults [1].

Maxillofacial plastic and reconstructive surgery encompass procedures — ranging from osteotomies and implant placement to trauma repair and tumor ablation — which place extraordinary regenerative demands on both hard and soft tissues [7]. Postoperative malnutrition is common: intermaxillary fixation, pain, xerostomia, and comorbid dysphagia limit oral intake at precisely the moment when protein and micronutrient requirements peak [8]. While dietary essential amino acids have been recognized as critical for collagen synthesis and immune competence, the contribution of nonessential amino acids has received comparatively little attention in clinical protocols [9]. This oversight is consequential: serine and glycine supply one-carbon units for nucleotide biosynthesis in proliferating fibroblasts [1], glutamine fuels macrophage activation and fibroblast collagen secretion [10, 11], arginine is the substrate for nitric-oxide-mediated vasodilation [12], and cysteine underpins glutathione synthesis, buffering the oxidative burst characteristic of the inflammatory phase of wound healing [13]. Aged bone, already compromised by osteoporosis, is particularly sensitive to serine-one-carbon insufficiency, which impairs osteoblast differentiation and mineralization [9, 14].

The present narrative review synthesizes mechanistic and translational evidence to answer three interconnected questions:

1. How does aging remodel the endogenous synthesis of key NEAAs? We map enzyme expression, co-factor availability, and hormonal regulation across the serine/glycine, glutamate/glutamine, aspartate/asparagine, arginine/citrulline, and cysteine/trans-sulfuration branches.
2. What are the consequences for craniofacial wound and bone healing? We relate pathway-specific deficits to each phase of healing — hemostasis, inflammation, proliferation, and remodeling — with an emphasis on osteogenesis in osteoporotic bone.
3. Can targeted nutritional or pharmacological interventions restore NEAA sufficiency and improve outcomes?

By weaving together these strands, we aim to provide oral and maxillofacial surgeons with a metabolic framework that informs perioperative nutrition, guides future clinical trials, and ultimately enhances patient recovery in the rapidly growing geriatric population.

## Main text

### Biochemical pathway overview — glucose as the carbon hub

**Fig. 1:**
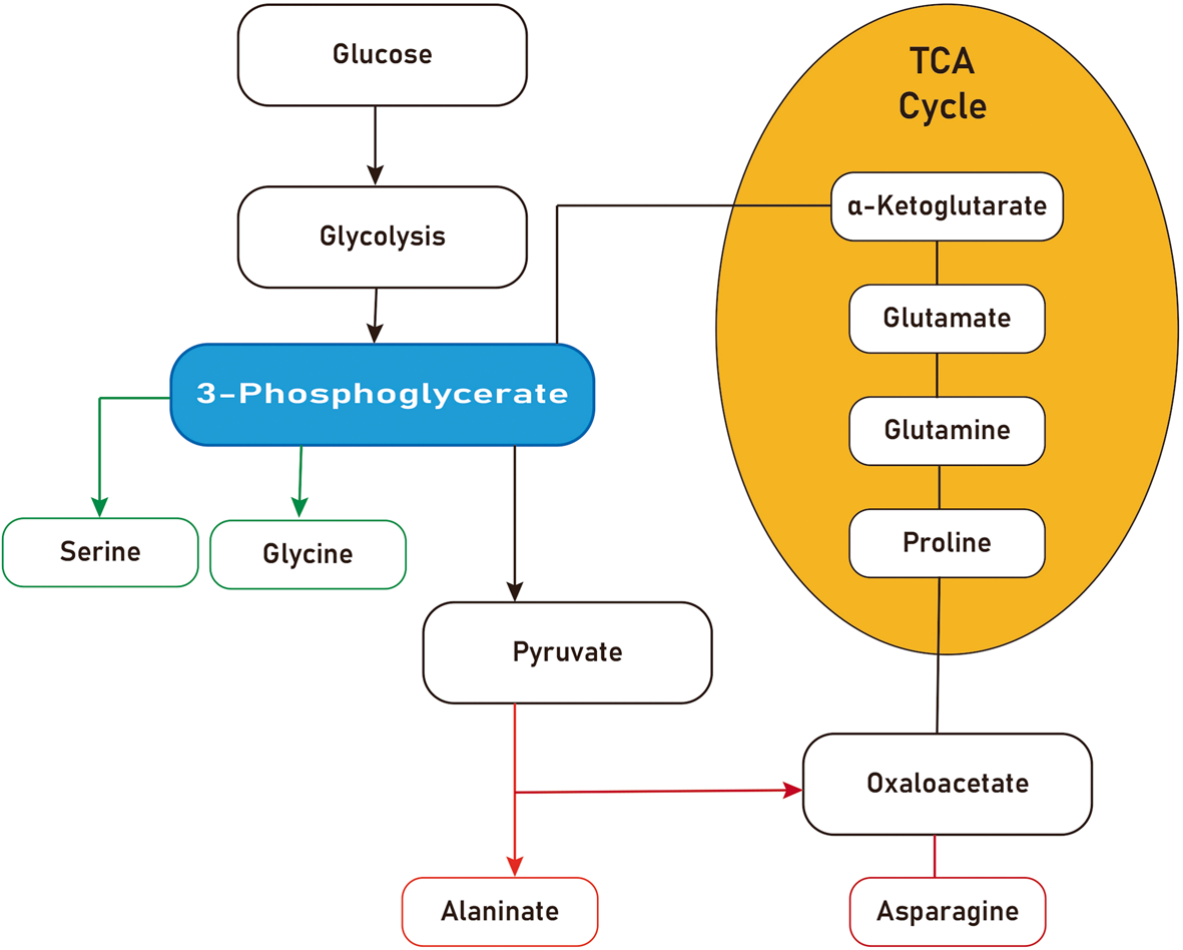
Color-coded schematic of glucose-derived NEAA pathways*.* Glycolysis intermediates (blue) feed the serine/glycine/cysteine branch (green), the pyruvate-alanine shuttle (red), and the TCA cycle (orange), which in turn supplies *α*-ketoglutarate and oxaloacetate branches. Dashed arrows denote multi-step conversions

Glucose catabolism provides carbon skeletons that feed directly into the biosynthesis of NEAAs, a process vital for regenerating tissues such as bone and connective tissue in maxillofacial repair, especially in geriatric patients. Glycolysis, for instance, supplies key intermediates that are diverted into amino acid production (Fig. 1). One prominent example is the glycolytic intermediate 3-phosphoglycerate, which is siphoned from the mid-glycolysis stream to generate L-serine through a three-step pathway [15]. First, 3-phosphoglycerate is oxidized by PHGDH (an NAD⁺-dependent enzyme) to form 3-phosphohydroxypyruvate [16]. Next, PSAT1 transaminates 3-phosphohydroxypyruvate, using glutamate as the nitrogen donor, to produce 3-phosphoserine while releasing *α*-ketoglutarate [17]. Finally, phosphoserine phosphatase (PSPH) hydrolyzes 3-phosphoserine to yield L-serine [17]. This de novo serine synthesis pathway is a major route by which glucose-derived carbons become amino acid building blocks in the cell [15]. L-Serine itself then serves as a metabolic node: it can be converted to glycine via serine hydroxymethyltransferase, which transfers a one-carbon unit from serine to tetrahydrofolate and produces glycine [18]. Serine also provides the carbon backbone for cysteine synthesis through the trans-sulfuration pathway: serine condenses with homocysteine (a derivative of methionine) in a reaction catalyzed by cystathionine *β*-synthase (CBS) to form cystathionine, which is then cleaved by cystathionine *γ*-lyase (CGL), yielding L-cysteine [19]. Notably, both CBS and CGL require vitamin B₆ (pyridoxal phosphate) as a cofactor, underscoring the cofactor dependence of amino acid biosynthetic enzymes [20]. Through these reactions, a single glycolytic intermediate (3-phosphoglycerate) is able to generate three important NEAAs — serine, glycine, and cysteine — which are essential for producing proteins and antioxidants [15]. This linkage is particularly relevant in wound healing, where rapidly dividing fibroblasts and osteoblasts require abundant serine for nucleotide synthesis and where cysteine availability can limit glutathione-mediated redox balance in regenerating tissues.

The serine synthesis pathway is finely regulated to balance supply with cellular demand. L-Serine itself can act as a feedback inhibitor of PHGDH in certain organisms, ensuring that excess serine slows its own production [21]. In humans, regulation occurs largely at the level of enzyme expression and activity in response to nutrient availability and stress. Under amino acid starvation or other stresses, the integrated stress response activates ATF4, a transcription factor that upregulates the genes encoding PHGDH, PSAT1, and PSPH, thereby enhancing serine output [22]. This ATF4-mediated induction is often triggered by the GCN2-eIF2α pathway when uncharged tRNAs accumulate (a sign of amino acid deprivation) [23]. Oncogenic signals also converge on the serine biosynthetic pathway: c-MYC, for example, increases flux through this pathway by elevating the transcription of its enzymes [22]. In hypoxic conditions, cells reprogram metabolism to maintain redox homeostasis; hypoxia-inducible factor-1α (HIF-1α) not only shifts cells to glycolysis but also diverts glycolytic intermediates into the serine/one-carbon pathway, increasing serine synthesis and downstream folate metabolism [24]. This hypoxia-driven serine flux helps generate NADPH and glutathione, protecting cells from oxidative damage [24]. Of note, the PHGDH step requires NAD⁺ as a cofactor [15], so the cellular NAD⁺/NADH ratio can influence serine production; a highly reduced state (low NAD⁺) as seen in ischemic or elderly tissues could constrain this pathway. In summary, the production of serine from glucose is dynamically controlled by feedback loops and transcriptional programs, linking it to the cell’s nutritional and oxygen status [22]. Clinically, this is significant because serine and glycine fuel the one-carbon pool needed for collagen cross-linking and DNA synthesis, while cysteine through glutathione supports cellular antioxidant defenses during healing (Table 1). Geriatric wound healing may benefit from ensuring these pathways remain active, as aging cells often exhibit attenuated ATF4 responses and redox imbalances that could limit serine availability and thus tissue repair.

**Table 1:** Transcriptional programs

Transcription factor	Primary NEAA targets	Activation cues	Age-related drift	Impact on cranio-maxillofacial healing
ATF4	*PHGDH*, *PSAT1*, *ASNS*, *CBS*	eIF2α phosphorylation (amino-acid deprivation, oxidative stress)	Blunted in resting muscle but hyper-inducible under chronic stress	Coordinates serine/one-carbon and asparagine supply for osteoblast proliferation and collagen secretion
c-MYC	Global amino-acid transporter genes; *GLS*, *GS*	Growth signals, Wnt/*β*-catenin	Declines with senescence	Drives glutamine and proline synthesis needed for rapid fibroblast expansion
HIF-1α	*PHGDH*, *P5 CS*, *GLUT1*	Hypoxia (common in surgical flaps)	Stabilization paradoxically increases in aged ischemic tissue	Promotes serine and proline production under low O₂, enhancing angiogenesis
NRF2	*SHMT2*, trans-sulfuration genes	ROS	Decreased nuclear translocation in elderly	Governs cysteine/glutathione axis, limiting oxidative damage at the fracture site

In parallel with the diversion of 3-phosphoglycerate to serine, the end product of glycolysis, pyruvate, serves as another crucial hub connecting carbohydrate metabolism to amino acid homeostasis. Alanine transaminase (ALT) — also known as glutamate-pyruvate transaminase — catalyzes the reversible transfer of an amino group from glutamate to pyruvate, forming L-alanine and *α*-ketoglutarate [15]. This reaction provides a direct link between glycolytic carbon and nitrogen cycling. In skeletal muscle, especially under catabolic stress such as intense exercise, fasting, or postsurgical recovery in the elderly, ALT activity is highly active to facilitate the glucose-alanine cycle (Cahill cycle) [25]. When muscle proteins are broken down, the resulting amino groups are transferred to pyruvate to produce alanine [26]. Alanine is released into the bloodstream and transported to the liver [27]. Once in the liver, the ALT reaction runs in reverse: alanine is transaminated with *α*-ketoglutarate to regenerate pyruvate and glutamate [28]. The pyruvate can then be used for gluconeogenesis — allowing the liver to synthesize glucose that can be sent back to tissues — while the glutamate enters the urea cycle for safe excretion of the nitrogen as urea [29]. This cycle is analogous to the Cori cycle (lactate-glucose cycle) but revolves around amino groups instead of lactate [30]. The Cahill cycle is vitally important for maintaining blood glucose and preventing ammonia toxicity during periods of stress [31]. In the context of wound healing and surgery, especially for geriatric patients who may have limited muscle reserves, this alanine shuttle helps spare muscle from excessive ammonia accumulation by exporting nitrogen to the liver as alanine. However, it also means that muscle protein provides crucial substrates for gluconeogenesis during times of caloric insufficiency or stress [30]. Thus, interventions that modulate muscle proteolysis or ALT activity could impact the nitrogen balance and energy supply in recovering patients. Maintaining moderate activity and good nutrition in postoperative elderly patients can support a healthy alanine cycle — providing energy to healing wounds without undue muscle wasting [32].

Within the mitochondria, glucose-derived carbons continue their journey through the TCA cycle, and the TCA intermediate *α*-ketoglutarate emerges as a central feeder into several NEAA biosynthetic pathways [15]. *α*-Ketoglutarate sits at the nexus of carbon–nitrogen metabolism: through transamination reactions, it is converted to glutamate, which in turn is the starting point for the glutamate family of amino acids (glutamine, proline, and arginine) as well as a key donor/acceptor of nitrogen in many metabolic processes [33]. One high-flux reaction is catalyzed by glutamate dehydrogenase, which can incorporate free ammonia into *α*-ketoglutarate to form glutamate [34]. This reaction, occurring in the mitochondrial matrix, effectively fixes excess nitrogen into an amino acid form [35]. Glutamate, once formed, serves as the direct precursor for glutamine [36]. The enzyme glutamine synthetase (GS) adds another ammonia (NH₄⁺) to glutamate’s side chain carboxyl group in an ATP-dependent reaction to produce L-glutamine [15]. Glutamine, the most abundant free amino acid in plasma [10], functions as a systemic nitrogen shuttle [37] and a key metabolic fuel for rapidly proliferating cells such as lymphocytes, enterocytes, and fibroblasts — particularly during immune activation and tissue repair [12, 38]. For example, activated immune cells in a surgical wound consume glutamine at high rates as it provides both energy and nitrogen for nucleotide synthesis [39]. In fact, glutamine is often considered “conditionally essential” in catabolic states because endogenous synthesis may not meet the heightened demand during severe stress or injury.

Glutamate can also be channeled into the production of proline, an amino acid especially important for collagen formation in healing bone and soft tissues [39]. The conversion of glutamate to proline begins with the activation of glutamate by *γ*-glutamyl kinase (the first enzymatic activity of the bifunctional pyrroline-5-carboxylate synthase, P5 CS), which uses ATP to phosphorylate glutamate to *γ*-glutamyl phosphate [15]. This intermediate is then reduced and cyclized by the second activity of P5 CS to form *Δ*^1^-pyrroline-5-carboxylate (P5 C). Finally, pyrroline-5-carboxylate reductase (PYCR) reduces P5 C to L-proline, using NADPH as the reducing agent [40]. These steps collectively require significant reducing power and energy, illustrating the dependency of anabolic synthesis on cellular redox and energy status. Proline is indispensable for the triple-helix structure of collagen — it constitutes a large fraction of collagen’s amino acid composition and confers stability to collagen fibers [40]. In a maxillofacial bone fracture or surgical wound, the local fibroblasts and osteoblasts must produce enormous quantities of collagen; thus, adequate proline synthesis from glucose can be rate-limiting for effective tissue repair. Elderly patients, who may have lower metabolic efficiency, could experience slower collagen deposition partly due to less robust proline biosynthesis, making nutritional support important (e.g., supplementation with proline-rich gelatin or vitamin C to aid proline hydroxylation) [41].

The metabolism of glutamate also connects with the urea cycle and arginine biosynthesis via the intermediate ornithine [42]. In mitochondria, ornithine aminotransferase (OAT) transfers an amino group from glutamate to the urea cycle amino acid ornithine, yielding glutamate semialdehyde and regenerating α-ketoglutarate [15]. This reaction links the glutamate-proline pathway with arginine metabolism: the produced glutamate semialdehyde can either proceed to proline or be used to regenerate ornithine, effectively creating an interchange between ornithine and proline pools [43]. Meanwhile, ornithine itself is a key substrate in the urea cycle, which is the primary route for arginine synthesis in the body [44]. In the liver, ornithine combines with carbamoyl phosphate (synthesized by carbamoyl phosphate synthetase I, an enzyme requiring two ATP molecules and allosteric activation by N-acetylglutamate) to form citrulline [16]. Citrulline is then exported to the cytosol and converted into argininosuccinate-by-argininosuccinate synthetase (ASS), a step that consumes ATP and uses aspartate as a nitrogen source [45]. Argininosuccinate is cleaved by argininosuccinate lyase to yield L-arginine [46]. Through these reactions, *α*-ketoglutarate ultimately enables the de novo production of arginine, another conditionally essential amino acid. Arginine produced in the urea cycle can be used for protein synthesis or cleaved by arginase to regenerate ornithine [47]. However, arginine has special roles in healing that go beyond incorporation into proteins [48]. It is the sole precursor for nitric oxide (NO) synthesis, a reaction carried out by nitric oxide synthases in endothelial cells and immune cells [15]. NO is a critical signaling molecule that, in wounds, promotes vasodilation, modulates inflammation, and even directs collagen deposition and remodeling [48]. Arginine is also a precursor for polyamines [49], which are required for cell proliferation. In the wound healing context, polyamines derived from arginine stimulate fibroblast proliferation and reepithelialization [50]. Because of these roles, arginine availability can significantly impact healing outcomes. Indeed, arginine levels often become insufficient during major stress, and arginine is considered conditionally essential in trauma and for elderly individuals [51]. Clinical studies have shown that arginine supplementation improves wound healing, evidenced by enhanced collagen deposition and wound strength [48]. For example, providing arginine-enriched nutrition to older patients with surgical wounds or pressure ulcers leads to increased hydroxyproline content in the wound and faster healing rates [48, 52]. This underscores how the metabolic pathway from glucose to arginine can be leveraged therapeutically to support tissue repair. In sum, the *α*-ketoglutarate node of the TCA cycle spawns multiple NEAAs — glutamate, glutamine, proline, and arginine — each with specific enzyme requirements and each contributing indispensably to cellular functions like immune response, structural protein synthesis, and signaling.

Another important glucose-derived intermediate feeding into amino acid biosynthesis is oxaloacetate, the end-point of the TCA cycle, which gives rise to the aspartate family of amino acids. Aspartate aminotransferase (AST), also known as glutamate–oxaloacetate transaminase (GOT), catalyzes the transfer of an amino group from glutamate to oxaloacetate, yielding L-aspartate and regenerating *α*-ketoglutarate [15]. Aspartate thus formed plays multifaceted roles in metabolism. It is a direct substrate for nucleotide biosynthesis — in particular, aspartate provides the carbon–nitrogen backbone in the de novo synthesis of pyrimidines and contributes a nitrogen in the purine synthesis pathway [53]. Aspartate is also crucial in the urea cycle: it condenses with citrulline to donate the second nitrogen for urea and form argininosuccinate [15]. Because of these functions, a sustained supply of aspartate is needed for proliferating cells [54] and for ammonia detoxification [55]. The generated aspartate can be further converted to asparagine, another NEAA, through the action of asparagine synthetase (ASNS) [55]. ASNS uses aspartate and glutamine as substrates: it activates aspartate with ATP to form an aspartyl-AMP intermediate and then uses the glutamine’s amide nitrogen to aminate this intermediate, producing L-asparagine and releasing glutamate [56]. This ATP-dependent amidation makes asparagine synthesis an energy-sensitive process. Regulation of asparagine levels is tightly linked to cellular stress responses; ATF4 (the same stress-responsive transcription factor that induces serine synthesis) also upregulates ASNS expression during amino acid deprivation or hypoxia, as asparagine becomes critical for cell survival under these conditions [57]. Asparagine itself functions more in a supportive role: while not a major structural amino acid, it influences protein synthesis and secretion and acts as an amino acid exchange factor in some contexts. Notably, in endothelial cells under hypoxic stress such as might occur in a poorly perfused wound or an ischemic bone graft, increased ASNS expression and asparagine production help cells cope by maintaining protein translation and redox balance [24]. Thus, oxaloacetate from glucose contributes to NEAA pools by yielding aspartate and asparagine, connecting carbohydrate metabolism to nucleotide synthesis and adaptive stress responses. For healing tissues, abundant aspartate ensures DNA synthesis for cell proliferation, and sufficient asparagine helps cells survive under the nutrient-limited, often hypoxic wound microenvironment [54].

All these pathways are orchestrated in an integrated manner, responding to the cell’s redox state and hormonal environment. A high NAD⁺/NADH ratio such as the oxidized state favors reactions like PHGDH and sustains flux through glycolysis into anabolic routes [15], whereas a reducing environment might slow such biosynthesis and divert pyruvate to lactate instead. NADPH, on the other hand, is the currency of reductive biosynthesis and antioxidant defense [58]; plentiful NADPH is needed for converting glutamate to proline [59] as well as for regenerating reduced glutathione from its oxidized form [60]. Cells maintain NADPH via the pentose phosphate pathway and serine-one-carbon metabolism which is enhanced under HIF-1α in hypoxia to boost NADPH production [24], thereby linking glucose metabolism to the redox requirements of amino acid synthesis. Hormonal signals profoundly influence these metabolic fluxes: insulin and insulin-like growth factor 1 (IGF-1) are anabolic hormones that promote glucose uptake and utilization and activate the mTOR pathway, a central regulator of cell growth and protein synthesis [61]. When insulin/IGF-1 signaling is robust as in well-fed, healthy conditions, mTOR activity is high, which encourages the use of available amino acids for protein assembly and upregulates enzymes in glycolysis and nutrient pathways [62]. mTOR also senses amino acid abundance directly; for example, sufficient intracellular leucine, arginine, and glutamine activate mTOR complex 1, which then enhances ribosomal biogenesis and mRNA translation, driving tissue growth and repair [63]. In contrast, during energy stress or nutrient scarcity common in postoperative or elderly individuals who might have anorexia or vascular insufficiency, AMP-activated protein kinase (AMPK) is activated [64]. AMPK shifts metabolism toward energy conservation [65] — it promotes catabolic processes like fatty acid oxidation to generate ATP [64] and inhibits anabolic processes that consume ATP, in part by downregulating mTOR [66]. An active AMPK and suppressed mTOR can lead to reduced protein and collagen synthesis and lower expression of some biosynthetic enzymes, potentially slowing wound healing [67, 68]. Moreover, hormonal changes with aging — such as insulin resistance, lower IGF-1 levels, and higher glucocorticoid levels — can tilt the balance toward a catabolic state, meaning geriatric patients often have a blunted anabolic response even with adequate nutrients [69, 70]. This can manifest as reduced efficiency in producing NEAAs and utilizing them for tissue repair. Therapeutically, approaches like improving insulin sensitivity through moderate exercise or pharmacology or providing growth factor supplementation might enhance anabolic metabolism in older patients to support healing [71, 72]. Additionally, the body has adaptive mechanisms to ensure critical amino acid supply during stress [73]: the rise in ATF4 during nutrient deprivation or in diabetes [74, 75] and aging-related oxidative stress can paradoxically boost certain amino acid biosynthetic pathways like serine, asparagine, and cysteine via trans-sulfuration to compensate for reduced dietary intake [76]. Thus, the interplay of redox state and hormonal signals finely tunes the activity of NEAA-producing pathways in accordance with the body’s needs and the healing process’s demands.

Finally, amino acid biosynthesis from glucose is not confined within single cells — it is a whole-body enterprise with organs cooperating to shuttle amino acid intermediates where they are needed. We have already seen the Cahill cycle as one example of an interorgan metabolic shuttle. Another is the intestinal-renal axis for arginine: the small intestine is a net producer of citrulline from glutamine and other precursors, especially after meals or during stress [77]. Enterocytes lack the complete urea cycle [42] and thus release citrulline into circulation rather than converting it fully to arginine [78]. The kidneys then take up citrulline [79] and, equipped with argininosuccinate synthetase and lyase, convert it to arginine [80], which is released into the bloodstream [81]. This two-organ pathway is crucial in states where arginine demand is high such as growth, injury, and sepsis; it effectively makes citrulline a carrier for regenerating arginine. In surgical patients and older adults, preserving gut and kidney function is important for this reason — they collectively maintain systemic arginine availability for immune cells and fibroblasts [79, 82]. Yet, another shuttle occurs in the central nervous system: the astrocyte-neuron glutamine cycle [83]. Here, neurotransmitter glutamate released from neurons is taken up by astroglial cells [84] and amidated to glutamine via GS [85]. The glutamine is then shuttled back to neurons, which can regenerate glutamate by glutaminase for neurotransmission [86]. While this cycle is specialized for neurotransmitter recycling, it exemplifies how one cell’s waste or byproduct can be another’s fuel. In the context of wound healing, a comparable principle is the recycling of metabolites within the wound milieu — immune cells and fibroblasts exchange arginine, ornithine, and citrullin e: for instance, activated macrophages metabolize arginine to ornithine via arginase to promote tissue repair, and neighboring fibroblasts can uptake ornithine to produce proline for collagen or convert it back to arginine if needed [48]. This cooperative metabolism ensures that key precursors for repair like proline and NO are optimally supplied. Overall, the integration of glucose metabolism with amino acid biosynthesis is a remarkable demonstration of metabolic flexibility and coordination. In maxillofacial plastic and reconstructive surgery, where healing of bone and soft tissue is paramount, these pathways provide the necessary amino acids for collagen synthesis (proline, glycine, arginine), cell proliferation (serine, aspartate, glutamine), and immune function (glutamine, arginine). Geriatric patients often have impairments in these metabolic integrations due to comorbidities or reduced physiological reserve, which can translate to slower wound healing. Recognizing the central role of glucose-driven NEAA biosynthesis has led to clinical nutritional strategies: for example, diets enriched in arginine, glutamine, and antioxidants are given to improve healing outcomes in elderly surgical patients [52]. Such interventions take advantage of the metabolic links described above — essentially “feeding” the pathways to ensure that a recovering patient’s cells have ample building blocks and regulatory molecules to mount an effective repair response. In conclusion, the flow of carbons from glucose into NEAAs —modulated by key enzymes and regulatory networks —underlies the capacity of tissues to heal and regenerate, illustrating why metabolic health is so closely tied to successful outcomes in maxillofacial reconstruction and wound recovery in the elderly.

### Age-related declines in NEAA metabolism impair wound healing phases

Aging adversely affects the metabolism of several NEAAs —notably serine, glycine, cysteine, proline, arginine, alanine, and asparagine —which are crucial for the overlapping phases of tissue healing [52]. Declines in these NEAA pathways compromise distinct aspects of wound repair, including the early inflammatory response, granulation tissue formation and angiogenesis, osteogenesis, mucosal regeneration, and neural reinnervation (Table 2). Below, we describe how age-related metabolic deficiencies in NEAAs lead to impaired healing, supported by mechanistic and clinical evidence.

**Table 2:** Age-related changes in nonessential amino acid biosynthesis

Pathway/key enzyme (rate limiter)	Typical age trend	Mechanistic notes & functional impact
Serine → glycine (PHGDH, PSAT1, PSPH)	↓In the liver, skeletal muscle, & endothelium	PHGDH transcription and NAD⁺ availability fall, serine/1‑C deficiency exacerbates hepatic steatosis and impairs muscle protein synthesis, and extracellular serine/glycine pools drop in old mice, blunting mTOR‑driven anabolism
Glutamate/glutamine (GS, GLT‑1)	Brain: ↑compensatory GS/GLT‑1; muscle & plasma: often ↓ or unchanged	Astrocytes up‑regulate glutamine synthetase and transporters to keep excitatory signaling in check, but whole‑body glutamine can fall during stress and sarcopenia
Aspartate → asparagine (ASNS)	Mixed; stress‑induced↑, baseline data-limited	ASNS is part of the integrated‑stress response; aging muscle shows blunted ISR, yet pharmacological or *β*‑adrenergic stimuli can still induce ASNS
Glutamate → proline/arginine (P5 CS, ASS/ASL)	↓Renal & intestinal flux → lower plasma arginine	Declining kidney function and intestinal citrulline output weaken the intestinal‑renal arginine axis; circulating Arg, Gly, and Orn falls with age
Serine + homocysteine → cysteine (CBS, CGL)	↓CBS/CGL activity → Less cysteine & glutathione	CBS expression falls in multiple tissues; impaired trans-sulfuration increases oxidative stress and homocysteine
Pyruvate ↔ alanine (ALT)	Depends on insulin sensitivity	Insulin resistance and lower muscle mass alter alanine cycling; data suggest altered flux rather than enzyme abundance

In the early inflammatory phase of healing, older individuals exhibit diminished capacity to control reactive oxygen species (ROS) and to mount robust immune cell responses [87]. Cysteine availability via the cystathionine *β*-synthase, CBS, trans-sulfuration pathway, and glutathione (GSH) levels is often reduced with age [88], weakening antioxidant defenses [89]. Indeed, wound tissues in aged animals have significantly lower GSH levels than in young adults, and experimental GSH depletion greatly reduces wound tensile strength [90]. Conversely, boosting GSH can rescue healing in impaired models [90], underscoring that normal cysteine/GSH metabolism is crucial for early wound integrity. Likewise, efficient leukocyte proliferation during inflammation depends on serine and glutamine metabolism. Serine supports nucleotide synthesis and the one-carbon cycle in rapidly dividing immune cells; T cells lacking the serine-responsive transcription factor ATF4 show defective proliferation, redox balance, and cytokine production [91]. In other words, serine-ATF4 pathways are needed to expand leukocytes for pathogen clearance. Glutamine, a conditionally essential NEAA during stress, is an equally important fuel for immune activation. Immune cells consume glutamine at rates comparable to or exceeding glucose, using it for proliferation, phagocytosis, and cytokine generation [10]. If aging or illness reduces glutamine availability, immune cell function in wounds is blunted — leading to prolonged inflammation or risk of infection. This has clinical relevance: in catabolic states or advanced age, plasma glutamine can become insufficient, reflecting muscle glutamine depletion and correlating with poor outcomes [92]. Thus, deficits in serine/ATF4 signaling and glutamine/glutaminase activity with age can impair leukocyte expansion and timely inflammatory resolution in wounds.

During the proliferative phase of healing, which includes granulation tissue formation, angiogenesis, and extracellular matrix deposition, declines in serine, glycine, and proline metabolism directly hinder collagen synthesis and neovascularization. Collagen, the predominant structural protein in healing tissues, is approximately one-third glycine, with proline and hydroxyproline together forming another ~ 23% [93]. Serine is a precursor for glycine via one-carbon metabolism, and all three NEAAs must be abundant for fibroblasts to synthesize collagen triple helices [1]. In aged wounds, lowered serine/glycine availability likely contributes to reduced collagen deposition and inferior tensile strength of the scar. Experimental models demonstrate that depleting GSH leads to weaker wound breaking strength [90]. Additionally, proline deficiency syndromes are known to cause impaired wound healing [93], highlighting the general principle that inadequate NEAA supply limits matrix production. Aging also attenuates angiogenesis and tissue perfusion during healing, in part due to arginine deficiencies [79, 94]. Arginine is the sole precursor for NO, a vasodilator and signaling molecule required for proper wound healing [79]. NO promotes inflammatory cell recruitment, stimulates endothelial and fibroblast activity, and is essential for new blood vessel ingrowth and perfusion of the regenerating tissue [95]. Normally, inducible NO synthase 2 is upregulated early in healing to produce NO from arginine, and this is a prerequisite for a normal inflammatory and granulation phase [95]. In stress or age, however, arginine bioavailability often becomes rate-limited [79]: arginase activity is increased, and renal conversion of citrulline to arginine is reduced [96]. As a result, tissue arginine levels drop, and NO production is impaired [95]. Clinical studies of fracture healing show that patients with atrophic nonunions have markedly lower arginine concentrations at the fracture site than patients with normal healing [95]. This regional arginine deficiency is thought to cause insufficient NO signaling for angiogenesis and osteogenesis, contributing to non-healing [95]. Preclinical data support this: supplemental arginine boosts local blood supply, angiogenesis, and collagen deposition in healing bone defects [97]. Thus, age-related decreases in arginine availability which can be exacerbated by insulin resistance or malnutrition can limit NO-mediated perfusion of wounds and the vascularization of repair tissue (Table 3).

**Table 3:** Osteogenesis and callus consolidation

Pathway	Age-related drift	Consequence for bone healing
Serine → collagen I & phosphatidyl-serine	Reduced serine biosynthesis and utilization in osteoblasts due to aging-associated declines in PHGDH and NAD⁺ levels	Impaired osteoid formation and delayed mineralization due to insufficient collagen matrix and phospholipid nucleation
Cysteine → glutathione	Decreased trans-sulfuration enzyme activity (CBS/CGL) leads to diminished glutathione synthesis in osteogenic cells	Elevated oxidative stress promotes osteoblast apoptosis and reduces differentiation capacity
D-Serine	Lower osteoblast-derived D-serine with age reduces extracellular pool and NMDA receptor signaling	Increased osteoclast activity and resorption at fracture sites; contributes to cortical porosity
Proline → hydroxyproline	Age-related decline in collagen cross-linking efficiency and hydroxyproline incorporation	Weakened collagen network, increased microcrack formation, reduced mechanical strength of healing bone

**Fig. 2:**
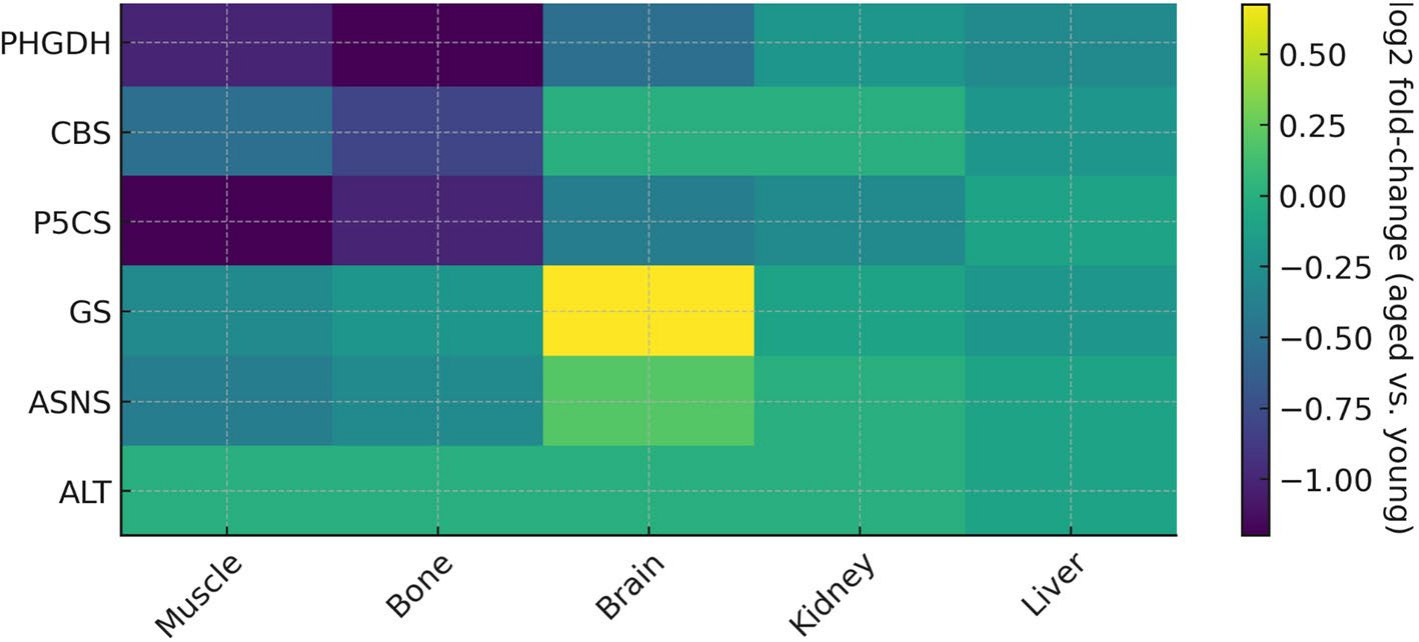
Tissue-resolved remodeling of NEAA pathway enzymes with aging. Heatmap showing log₂ fold-changes in the expression of key nonessential amino acid (NEAA) biosynthetic enzymes in aged versus young tissues. Values represent transcriptomic shifts for seven pathway-limiting enzymes — PHGDH, CBS, P5 CS, GS, ASNS, and ALT — across five organs: muscle, bone, brain, kidney, and liver. Negative values (purple/blue) indicate age-related downregulation; positive values (green/yellow) indicate upregulation. The data highlight tissue-specific remodeling, including marked declines in PHGDH and P5 CS in muscle and bone, and selective upregulation of GS and ASNS in the aging brain

Compromised NEAA metabolism in the elderly also affects tissue-specific regeneration processes such as bone repair, mucosal healing, and nerve reinnervation (Fig. 2). In bone healing, adequate arginine and proline metabolism are particularly important [95]. Arginine-derived NO not only promotes angiogenesis in the fracture callus but also stimulates osteoblasts and osteoclasts to orchestrate bone remodeling [97]. Experiments show arginine supplementation increases osteoblast activity, callus formation, and bone mineralization in fracture models [97]. Proline and glycine, meanwhile, are required for synthesizing the collagen-rich osteoid matrix in bone. If serine, glycine, and proline production are blunted with age, the bone callus may be deficient in collagen content and mechanical strength. In a metabolomic profiling of human fracture healing, atrophic nonunions were found to have a broad depletion of amino acids within the local callus with significantly lower serine, glycine, and others relative to healing fractures [95]. Additionally, the metabolic balance of glutamine and glutamate is altered: nonhealing callus tissues showed a trend toward elevated glutamate levels, suggesting increased glutaminolysis to meet the heightened demand for proline and other biosynthetic precursors [95]. In other words, aged or compromised bone may be “draining” glutamine to produce glutamate and proline for repair, yet still not keeping pace with demand [95]. These findings reinforce that multi-tissue metabolic deficits — low serine/glycine and arginine, with dysregulated glutamine-glutamate cycling — underlie the impaired bone healing often seen in older patients [4, 14, 98]. Similarly, mucosal regeneration is hampered by NEAA decline. Glutamine is the principal fuel for intestinal epithelial cells and lymphocytes in gut-associated lymphoid tissue, and it drives mucosal protein synthesis and regeneration. In advanced age, a drop in plasma glutamine and muscle stores leads to gut mucosal atrophy [92]. Animal studies have shown that glutamine supplementation in aged rats can increase intestinal mass and villus height, effectively reversing age-related mucosal thinning [92]. This demonstrates that maintaining glutamine metabolism is critical for mucosal healing and barrier function in the elderly. Other NEAAs, like arginine and asparagine, also contribute to mucosal and cutaneous repair by supporting cell proliferation and protein synthesis, and their deficiency in frail older adults may further impair tissue turnover [79, 99].

Finally, neural reinnervation after injury — an often-overlooked aspect of wound healing in sensory organs or surgical sites — may be delayed in aging due to declines in D-amino acid metabolism. D-Serine, the enantiomer of serine, acts as an essential co-agonist at NMDA-type glutamate receptors, which are involved in nerve growth and synaptic plasticity [100]. D-Serine, synthesized by serine racemase in both astrocytes and neurons, serves as a co-agonist at NMDA-type glutamate receptors, playing a pivotal role in modulating synaptic plasticity and possibly influencing neuronal recovery processes, although its role in axonal regeneration remains to be fully defined [101]. With advancing age, serine racemase expression and D-serine availability decline in the central nervous system [102]. This leads to reduced NMDA receptor activation, which can hamper neural recovery. For example, the regeneration of an injured trigeminal nerve after oral surgery may be slower in an elderly patient partly because lower D-serine impairs NMDA-receptor-dependent axon sprouting and remyelination. Age-related D-serine loss has been implicated in delayed functional recovery and synaptic reorganization in other contexts of neural injury [102]. Therefore, interventions that sustain D-serine levels or NMDA signaling might improve neural reinnervation in older adults. More broadly, the decline in serine and glutamine metabolism with age also affects Schwann cells and macrophages in nerve injury sites, cells that require these amino acids for repair processes [103, 104].

In summary, advanced age is associated with a constellation of metabolic shortfalls in NEAAs that collectively impair the healing process. From the diminished ROS buffering due to low cysteine/GSH [90], to slower immune cell proliferation from serine and glutamine paucity [10, 91], to weaker collagen scaffolding from glycine and proline shortfalls [93], and reduced perfusion and tissue anabolism from arginine/NO insufficiency [95] — the elderly healing environment is at a distinct disadvantage. These deficits extend to specialized tissues, with poor amino-acid support for bone formation [95], mucosal repair [92], and nerve recovery [102]. Multi-tissue metabolomic studies reinforce that healing tissues in older or malnourished individuals are depleted of key NEAAs (serine, glycine, arginine) and show imbalances in amino acid utilization [95]. Therapeutically, this suggests that targeted nutritional or pharmacologic supplementation of NEAAs may help restore the metabolic environment toward that of younger adults and improve healing outcomes in the geriatric population [95]. Robust clinical trials are needed to test whether correcting these age-related amino acid deficiencies can consistently speed up wound closure, enhance bone union, and promote neural regeneration in older patients.

### Rationale for amino acid supplementation in geriatric osteoporotic patients

Geriatric and osteoporotic patients often experience diminished bone healing capacity due to age-related sarcopenia, nutritional deficiencies, and impaired anabolic signaling. In aging, protein undernutrition and anabolic resistance contribute to osteoporosis and sarcopenia [105, 106]. Ensuring an adequate supply of amino acids is crucial because they serve not only as building blocks for bone matrix and muscle but also as signaling molecules that regulate bone turnover. Contrary to outdated concerns that high protein intake might induce bone loss, recent evidence shows that higher dietary protein (1.2–1.5 g/kg/day) correlates with improved bone mineral density by providing collagen precursors [105]. Essential amino acids (EAAs) have positive effects on bone cells: for example, lysine, threonine, methionine, tryptophan, and isoleucine have been shown to stimulate osteoblast proliferation/differentiation and simultaneously reduce osteoclast activity [105, 107]. Muscle and bone health are strongly interconnected — improvements in muscle mass and function relieve skeletal loading stress and enhance stability [108]. Therefore, nutritional strategies that preserve lean mass can secondarily benefit bone integrity. Unfortunately, up to 50% of older surgical patients are malnourished, which is associated with higher complication rates and poorer healing [106, 109]. This creates a compelling rationale to supplement key amino acids in the perioperative period for osteoporotic patients: doing so may counteract deficits in collagen synthesis and cell proliferation, activate anabolic pathways, and ultimately promote better bone repair and remodeling. In summary, targeted amino acid supplementation addresses an important reversible factor in age-compromised healing by supplying both the raw materials and metabolic signals needed for bone regeneration.

#### Therapeutic role of sericin and serine isoforms in bone metabolism

One promising intervention is sericin, a silk-derived protein particularly rich in L-serine (~ 30% of its amino acid content) [9]. Sericin has a unique dual action on bone metabolism through its breakdown products L-serine and D-serine. When ingested, sericin is hydrolyzed to free amino acids; the abundant L-serine can be partially converted to D-serine by the enzyme serine racemase in vivo [9]. These serine isoforms play complementary roles in bone homeostasis. While direct evidence of L-serine driving osteoblast differentiation is limited, its support of anabolic pathways such as mTOR signaling suggests that it may enhance osteoblast function and bone formation indirectly by promoting mitochondrial activity and biosynthetic capacity during late-stage osteoblast maturation [110]. D-Serine inhibits osteoclast development and bone resorption [9, 111]. In cellular studies, supplementation with L-serine significantly upregulates osteogenic markers such as alkaline phosphatase, Runx2, osterix, and type 1 collagen) in osteoblasts, indicating accelerated differentiation, whereas D-serine exposure suppresses osteoclast markers like cathepsin K, indicating reduced bone resorptive activity [9]. Animal models provide further support — diets enriched with sericin lead to elevated circulating D-serine and a measurable increase in trabecular bone mass in osteoporotic mice [9]. In addition, L-serine-enriched bone grafts have been shown to promote greater new bone formation, potentially by supporting osteoblast metabolic activity and matrix production at the graft site [112]. Through this coupled mechanism, sericin helps rebalance skeletal remodeling in favor of bone gain. Notably, sericin is also reported to possess antioxidant [113] and anti-inflammatory properties [114], which could indirectly benefit bone by mitigating oxidative stress in the aging skeletal microenvironment.

In practical terms, sericin can be delivered as an oral supplement as a protein isolate or “silk protein” powder to leverage its systemic effects. Oral sericin supplementation in experimental settings has improved bone density without significant adverse effects, though human clinical data are still needed [9]. In osteoporotic or frail patients, a proposed regimen is to use sericin as a nutraceutical supplement providing a few grams per day of additional protein, which translates to roughly 1–1.5 g of L-serine yield — enough to measurably raise plasma L-serine/D-serine levels [9]. This could be taken as a daily drink or added to meals in the weeks before and after surgery to support bone anabolism.

Beyond oral use, sericin also shows therapeutic potential as a topical or local treatment in bone and wound healing. Its biocompatibility and gel-forming properties allow it to be incorporated into scaffolds, membranes, or dressings applied at surgical sites. Sericin-based wound dressings have been demonstrated to accelerate wound closure, reduce scar formation, and enhance tissue regeneration in vivo [115]. In the context of bone surgery, sericin-enriched biomaterials can improve the healing milieu. Sericin promotes adhesion and proliferation of osteoblast-lineage cells on scaffolds [115], and it can stimulate local collagen production and mineralization [116]. In vitro, sericin-containing hydroxyapatite composites induced greater osteogenic differentiation of bone marrow stem cells and more robust formation of mineral crystals compared to controls [115]. The amino acid composition of sericin (rich in serine, glutamate, aspartate, etc.) appears to aid mineralization by chelating calcium/phosphate and by upregulating collagen matrix deposition [117, 118]. These findings suggest that a surgical site treated with a sericin-infused scaffold or gel might heal faster and with higher-quality bone [119]. Although clinical studies of topical sericin applications in orthopedics or maxillofacial surgery are currently lacking, the concept aligns with existing regenerative strategies. In principle, a sericin-based sponge could serve as a biodegradable matrix within extraction sockets or fracture sites, releasing L-serine locally to support healing [112] — a hypothesis that warrants further investigation. Overall, sericin offers a multifaceted therapeutic profile (nutritional, cellular, and biochemical) making it a compelling adjunct for geriatric bone healing, with the L- and D-serine components specifically driving osteoblast upregulation and osteoclast inhibition [9].

#### Oral amino acid supplementation strategies

In addition to sericin, several specific amino acids or amino acid combinations have been identified as beneficial for bone and collagen synthesis in the elderly. Glycine and proline are two such amino acids central to collagen structure: every third residue in collagen is glycine, and proline constitutes a large fraction of the remainder [120]. Older adults may not synthesize enough glycine and proline to meet the heightened demand of tissue repair, effectively rendering them “conditionally essential” during periods of bone healing [121]. Research has shown that collagen production can be strongly limited by glycine availability [122]. For instance, articular chondrocytes in glycine-poor conditions produce significantly less collagen, and only when excess glycine is supplied does collagen synthesis increase substantially [123]. In that study, raising media glycine concentration led to a 60–75% persistent increase in collagen II production, a result that revealed a “severe glycine deficiency” for maximal collagen formation under standard conditions [123]. Proline supplementation also enhanced collagen synthesis, though its effect plateaued at lower concentrations, consistent with proline being required but perhaps more readily available or synthesized than glycine [123]. These findings underscore the importance of providing ample glycine and proline to support the organic bone matrix in healing patients. Clinically, this can be achieved by oral supplements such as collagen peptides or gelatine, which are rich in glycine, proline, and hydroxyproline. A typical regimen involves approximately 10 g of collagen hydrolysate daily, often consumed as a powder mixed into a beverage [124]. This dosage provides several grams of glycine and proline — amino acids integral to collagen structure. Clinical studies have linked such supplementation to improvements in joint health, including reduced pain and enhanced function, particularly in individuals with osteoarthritis [125]. While some research suggests potential benefits for bone mineral density, findings are mixed, and further studies are needed to confirm these effects.

Another key amino acid intervention is L-citrulline. Citrulline is not a direct component of proteins but serves as a precursor to L-arginine, which is crucial for NO production, polyamine synthesis, and other pathways involved in tissue repair [126]. In elderly individuals, oral citrulline has shown considerable promise in improving nutritional and functional status. A randomized trial in malnourished older patients (average age > 80) found that 10 g/day of citrulline for 3 weeks significantly increased amino acid availability, with gains in lean body mass and appendicular muscle mass and a reduction in fat mass [127]. These anabolic effects suggest citrulline helps overcome age-related resistance to muscle protein synthesis, likely by boosting arginine and NO-dependent blood perfusion to muscle and bone. Improved muscle function can indirectly benefit bone by enabling better exercise and load-bearing [108]. Even at lower doses (e.g., 3 g/day of citrulline malate for 6 weeks), citrulline has been shown to enhance gait speed in older adults [127], indicating better functional mobility. From a bone-healing perspective, arginine contributes to fracture repair by stimulating angiogenesis and collagen deposition in the callus; arginine-derived NO can increase local blood supply and modulate inflammation in early healing [97]. Some animal studies support that arginine supplementation accelerates fracture healing and even inhibits osteoclast-mediated bone loss via NO signaling [128]. Therefore, including citrulline in a perioperative supplement regimen can aid an elderly patient’s circulatory support and anabolic drive needed for bone regeneration. Citrulline is generally well-tolerated, making it suitable for frail patients. It can be given as a standalone powder or combined with other supplements; notably, citrulline pairs well with exercise interventions to further improve outcomes [127].

N-acetylcysteine (NAC) is another supplement with a distinct therapeutic role: it bolsters the antioxidant capacity [129] and influences cell signaling in bone [130]. NAC provides cysteine, a rate-limiting precursor for glutathione, thereby mitigating oxidative stress which is often elevated in aged or osteoporotic bone tissue [131]. Beyond its antioxidant role, NAC has been found to enhance osteogenic processes. In vitro, NAC can accelerate the differentiation of osteoblasts and protect osteoblast-lineage cells from oxidative apoptosis [132]. In vivo, systemic NAC supplementation has yielded impressive results in models of impaired bone healing. A recent study in mice with age-related osteopenia showed that providing NAC (40 mM in drinking water) for 4 weeks increased bone mass accrual and significantly improved bone defect healing [132]. Mechanistically, NAC helped reactivate Wnt-associated osteoblast activity in those mice and simultaneously reduced oxidative stress, cellular senescence, and inflammation in the bone marrow niche [132]. Essentially, NAC rejuvenated the bone microenvironment: it preserved bone marrow stem cell function and promoted the formation of new bone in conditions where healing would normally be compromised [133]. Clinically, NAC (commonly 600–1200 mg/day orally) is a safe, inexpensive drug used for other indications such as mucolysis and hepatoprotection, and its application in bone healing is an emerging concept [132, 134]. It could be used prophylactically in older surgical patients to precondition their cells against oxidative damage [134]. Moreover, NAC can be delivered locally; for example, loading NAC into a collagen sponge or bone graft material has been shown to enhance local bone regeneration by preventing oxidative stress in the graft area [135]. Such an approach might be particularly useful in smokers or diabetic patients with high oxidative burdens. By combining NAC with amino acid supplements like glycine, one targets both the antioxidant system and the structural protein synthesis —a synergy that has been reported to improve strength and mitochondrial function in elderly humans [136]. Taken together, oral supplementation strategies for the geriatric osteoporotic patient would typically include a balanced mix of these components: a protein base to provide general EAAs including glycine/proline, specialized amino acids like L-citrulline for anabolic and circulatory support, NAC for redox modulation, and sericin for its serine content and unique bone-targeted effects. Each plays a role in optimizing the milieu for bone repair.

#### Modulation of anabolic pathways

Amino acid supplementation exerts its benefits not only by supplying substrates but also by modulating critical metabolic pathways that govern cell growth and tissue regeneration. Central among these is the mTOR pathway (mechanistic Target of Rapamycin), a nutrient-sensing signaling cascade that promotes protein synthesis and anabolic metabolism in response to amino acids. In osteoblasts, mTOR activation is associated with enhanced differentiation and function [105]. Sufficient levels of key amino acids — particularly leucine — activate mTORC1, leading to increased osteoblast protein synthesis and bone matrix production [137]. Conversely, in states of amino acid deprivation, cells downregulate mTOR activity and enter a catabolic or quiescent state [138]. One way this is mediated is through the general control nonderepressible 2 (GCN2) kinase, an eIF2α kinase that acts as an amino acid sensor [139]. When any essential amino acid is scarce, uncharged tRNAs accumulate and activate GCN2, which then triggers the integrated stress response and elevates ATF4, a transcription factor [76]. ATF4 helps the cell adapt by upregulating amino acid transporters and other survival genes, but chronically high ATF4 can inhibit proliferation and protein synthesis [140]. In the context of bone, GCN2 plays a nuanced role. Surprisingly, baseline GCN2 activity appears to be required for osteoprogenitor cells to proliferate robustly: in a mouse knockout model, loss of GCN2 led to significantly lower osteoblast numbers and reduced bone mass because skeletal stem cells could not effectively ramp up amino acid uptake for growth [141]. This suggests that a proper balance is needed — amino acid sufficiency keeps GCN2 activity in a beneficial range that supports osteoblast precursor proliferation via ATF4-driven nutrient uptake, whereas an insufficiency would over-activate GCN2’s stress response and stall cell cycling [141]. The practical implication is that by providing abundant amino acids, we aim to prevent pathologic amino acid shortfalls that would excessively engage GCN2 and dampen mTOR. Instead, ample amino acids favor sustained mTOR signaling for bone formation while allowing GCN2 to play its supportive role without inducing a full stress response [142]. Leucine-rich supplements can be particularly effective at raising mTOR activity in aging muscle and bone, thereby overcoming anabolic resistance [143], and citrulline-derived arginine can further stimulate mTOR via insulin and growth hormone secretion [128].

Another pathway of interest is the serine biosynthesis axis, centered on the phosphoglycerate dehydrogenase (PHGDH), which diverts glycolytic intermediates to produce serine [144]. Rapidly proliferating cells often upregulate PHGDH to meet the demand for serine and related metabolites [145]. In aging, defects in serine availability or synthesis can contribute to cellular senescence. In fact, experimentally inhibiting PHGDH in human dental pulp stem cells triggers premature cellular senescence [1], highlighting that an adequate serine supply is essential to maintain the proliferative capacity of somatic stem cells. Osteoblast-lineage cells likely face a similar requirement for serine to support collagen production and ATP generation; thus, supplementing serine may alleviate the need for excessive de novo serine synthesis and prevent the metabolic strain that can lead to cell cycle arrest [146]. On the flip side, osteoclasts rely on serine metabolism for their bone-resorbing function: deletion of PHGDH in osteoclast progenitors was shown to impair osteoclastogenesis and resulted in increased bone mass in mice [14]. This finding suggests that modulating serine pathways could tilt the balance of remodeling —ample serine for osteoblasts to build bone, but potentially limiting osteoclast activity if serine metabolism in those cells is disrupted. While we would not deliberately inhibit PHGDH in patients as that could have wide-ranging effects, understanding this biology reinforces why serine is a cornerstone of bone metabolism. Supplying L-serine exogenously may support osteoblastic cells and allow some conversion to D-serine, which, as noted, can directly curb osteoclast-mediated resorption [9]. In summary, interventions like sericin, glycine/proline, citrulline, and NAC favorably adjust the mTOR-GCN2-PHGDH network: they ensure mTOR has the amino acid signals to drive anabolism, prevent hyperactivation of GCN2 that would occur in nutrient deficits, and provide serine and cysteine to bypass bottlenecks in cellular biosynthesis. These molecular effects underpin the observed improvements in osteogenic activity and healing outcomes with amino acid supplementation.

#### Perioperative nutritional and lifestyle protocols

Optimizing bone healing in geriatric patients requires a holistic approach that goes beyond individual supplements. Perioperative lifestyle protocols are designed to create an optimal physiologic environment for healing during the critical periods before and after surgery. A foundation of this approach is ensuring sufficient overall nutrition and protein intake. Where possible, patients should receive a balanced diet providing at least 1.2 g/kg/day of high-quality protein in the weeks surrounding a procedure [105]. This dietary protein provides a broad base of essential amino acids to support wound healing and immune function, on top of which targeted amino acid supplements act synergistically. In malnourished or frail elders, initiating oral nutritional supplementation (ONS) is often necessary: energy-dense, protein-rich shakes or formulas can prevent weight loss and have been shown to improve postoperative functional outcomes in older adults [147]. Many commercially available ONS can be enhanced by the addition of the specific amino acids discussed.

Physical activity is the other pillar of perioperative care. Exercise protocols — tailored to the patient’s capacity — should be implemented pre- and postoperatively to stimulate muscle and bone [148]. Even low-intensity resistance exercises or weight-bearing activities can signal the musculoskeletal system to utilize the extra amino acids for tissue building rather than letting them be wasted. There is evidence that exercise combined with protein or amino acid supplementation produces superior gains in muscle mass and strength in the elderly than either alone [127]. In a pre-surgery setting, a short “prehabilitation” program of resistance training can improve a patient’s baseline fitness, which translates to better recovery and fewer complications [149]. Improved muscle strength from such programs also aids in fall prevention and mobility, reducing the risk of periprosthetic fractures or implant failures after orthopedic and maxillofacial procedures. Additionally, patients should be counseled on lifestyle factors that directly affect bone healing: smoking cessation is critical (as tobacco use delays healing and impairs blood flow to bones) [150], and alcohol consumption should be limited because chronic alcohol can weaken bone and immune responses [151]. Adequate sleep and stress reduction may also benefit hormonal balance that influences healing. Ensuring vitamin and mineral sufficiency is another aspect — calcium and vitamin D are obvious requirements for bone regeneration [152], and vitamin D deficiency is common in the elderly [153]. Patients should have their vitamin D levels checked and corrected to at least the normal range (30 ng/mL 25(OH)D or above) prior to elective bone-related surgeries, as vitamin D is necessary for calcium absorption and osteoblastic activity [154]. Other micronutrients like vitamin K, magnesium, and zinc play roles in bone health and should be provided either via diet or supplementation as needed. By addressing these lifestyle and nutritional factors in tandem with amino acid therapy, the perioperative protocol creates an anabolic, low-stress environment optimal for bone repair. For example, a comprehensive protocol might involve the following: a nutrition consult 4–6 weeks pre-surgery to start protein + amino acid supplementation and correct deficiencies, an exercise regimen to follow 2–3 times a week, and habit counseling (smoking/alcohol) well in advance of the procedure [155]. This proactive optimization has been linked to reductions in postoperative complications, shorter hospital stays, and improved rehabilitation in geriatric surgical patients.

#### Integration into clinical workflows: screening, implementation, and monitoring

Translating these strategies into routine clinical practice involves establishing a clear protocol for screening, supplementing, and monitoring patients. The first step is screening: identifying patients who would benefit most from amino acid and nutritional intervention. All older patients (generally > 65 years) or those with known osteoporosis/low bone density should undergo a nutritional risk assessment when planning major dental or orthopedic procedures [156]. Tools such as the Mini Nutritional Assessment (MNA) or serum albumin levels can objectively gauge nutritional status; indeed, low albumin and recent weight loss are strong predictors of poor postoperative outcomes in the elderly [157]. In addition to general nutrition, specific screening for sarcopenia and frailty is advised — simple measures like grip strength, gait speed, or questionnaires can unmask deficits in muscle function that imply anabolic insufficiency. Bone health should be evaluated via bone mineral density or fracture risk scores if not recently done, as this will inform the intensity of intervention needed [158]. Baseline laboratory workups might include 25(OH) vitamin D, calcium, renal function, and markers of inflammation or malnutrition (CRP, prealbumin) [159]. Once at-risk patients are identified, the healthcare team can initiate the supplementation protocol well ahead of surgery or during early treatment planning for fractures.

Implementation of supplementation requires coordination and patient education. Typically, a dietitian or clinical nutritionist would be involved to tailor the regimen to the individual’s needs and tolerances. For example, a frail 80-year-old woman scheduled for a dental implant could be started on the following: a whey protein supplement (to bring total protein intake to ~ 1.2 g/kg), plus a daily collagen peptide powder (10 g providing glycine/proline), plus L-citrulline (5 g, which can be given in the same drink), plus NAC (600 mg, perhaps as an effervescent tablet), and a multivitamin with vitamins D and K [160–162]. If sericin supplementation is available (as a capsule or powder), that could be added at a dose supplying ~ 3 g of sericin (yielding about 1-g L-serine). This regimen would be explained to the patient and possibly their caregiver, emphasizing compliance and how each component contributes to healing. Written instructions and schedules (e.g., “take citrulline before breakfast, collagen in the afternoon snack, NAC in the evening”) improve adherence. The supplementation should begin preoperatively (often 2–4 weeks prior) to build up nutrient reserves and continue through the postoperative period until initial healing milestones are passed (for bone, this might be 6–12-week post-surgery, corresponding to early consolidation of bone) [163]. In surgical inpatient settings, these supplements can be integrated into hospital meal plans or as medical nutrition therapy [164]. It is important to also integrate these nutritional measures with standard clinical pathways: patients with severe osteoporosis should concurrently be managed with appropriate medications, and physical therapy should continue to work on mobilization which synergizes with the nutritional gains.

Continuous monitoring is the third component of the workflow. Clinicians should regularly assess the patient’s progress and nutritional status throughout the perioperative course. This includes monitoring weight and muscle mass, checking wound healing and bone healing at follow-up visits, and tracking any functional improvements. For bone healing, periodic imaging (such as X-rays at 4, 8, and 12 weeks after a fracture repair or orthognathic surgery) can provide objective evidence of callus formation or graft integration. If healing appears delayed, one might extend the duration of supplementation or investigate other contributing factors like poor blood sugar control or undiagnosed endocrine issues. Some biochemical monitoring might be employed in research settings — for example, measuring serum amino acid levels (serine, citrulline/arginine) [165] or bone turnover markers (P1 NP, CTX) to see if the interventions are producing the desired metabolic changes [166]. In practice, a more straightforward monitor is patient outcome: strength testing, time to recover mobility, or, in dentistry, the success of osseointegration of an implant at 3–6 months. It is also crucial to monitor for any adverse effects of supplements. Generally, the described amino acids are safe: however, high-dose protein or certain amino acids can cause digestive upset (e.g., L-arginine might cause mild diarrhea at 9 g for some [167], and NAC can cause nausea for a minority [168]). Renal function should be watched if protein intake is substantially increased, especially in patients with marginal kidney function, though studies have not shown harm up to 1.5 g/kg protein in elders with normal renal function [127]. By setting up scheduled follow-ups, the healthcare team can ensure the protocol is being adhered to and adjust as needed. Embedding these steps into standard care pathways will facilitate adoption. Ultimately, integration into clinical workflow means the surgeon, geriatrician, dietitian, and possibly physical therapist collaborate, using screening tools to identify needs, providing the supplements and lifestyle guidance as a standard part of the care, and using objective healing and functional outcomes to measure success.

### Research gaps and future directions

While the above strategies are grounded in current scientific understanding, there remain important research gaps before they can be universally recommended. Most notably, large-scale clinical trials in the target population are lacking. Many of the benefits of sericin, glycine-proline supplementation, citrulline, and NAC have been demonstrated in animal models or small human studies, but we do not yet have randomized controlled trials showing that a comprehensive amino acid supplementation protocol improves fracture healing or surgical outcomes in osteoporotic geriatric patients. For example, the dual effect of sericin on osteoblasts and osteoclasts has been shown in rodents [9], but clinical studies are needed to confirm the efficacy and safety of sericin as an osteoporosis or bone-healing treatment in humans. The optimal dosage and formulation of sericin for human use remain undefined, as does the question of how its conversion to D-serine varies between individuals. Further exploration of the interaction between L-serine and D-serine in patients is warranted — their relative levels and conversion rates might influence treatment outcomes, and excess D-serine could have off-target effects since D-serine also acts as a neurotransmitter [169]. Another gap is understanding how these supplements interact with standard osteoporosis medications or other supplements: for instance, if a patient is on bisphosphonates or denosumab, would adding serine or citrulline confer additional benefit, or could there be diminishing returns? It will be important to test combination therapy. Long-term safety data is another consideration. Although amino acids are natural nutrients, chronically administering high doses to older adults has not been extensively studied; issues like metabolic overload, altered renal handling, or interactions with other metabolic pathways need attention (Table 4).

**Table 4:** Pharmacologic pathway modulation

Target	Agent	Expected effect	Status
PHGDH activation	NAD⁺ boosters (nicotinamide riboside 300 mg day^−1^)	Revives serine biosynthesis	Phase II trials in sarcopenia [170]
NRF2 induction	Sulforaphane (broccoli-sprout extract 30 mg)	Up-regulates CBS & CGL → cysteine/glutathione	Over-the-counter nutraceutical [171, 172]
GCN2 fine-tuning	Timed leucine supplementation (3 g pre-sleep)	Enhances collagen peptide synthesis via mTORC1 activation while preserving ISR-dependent NEAA gene expression	Small crossover trials show increased bone formation markers; further studies needed [173]
mTORC1 sensitization	Low-dose *β*-hydroxy *β*-methylbutyrate (1.5 g) with sericin	Amplifies translation of NEAA pathway mRNAs	Supported in frailty RCTs [174]

From a mechanistic standpoint, more research is needed to clarify the molecular pathways in human subjects: for example, does amino acid supplementation measurably suppress GCN2 activation or enhance mTOR signaling in patient bone biopsies or blood markers? Are there biomarkers such as an increase in serum D-serine or a decrease in urinary N-telopeptide if bone resorption is reduced that can serve as early indicators of efficacy? Understanding these will help refine protocols — one could imagine tailoring supplementation based on biomarker responses. Additionally, the topical application of amino acids like sericin and NAC in clinical practice raises questions as follows: what is the best delivery vehicle? How much of an oral supplement’s effect can be achieved with local delivery? The field of bone tissue engineering is exploring sericin-based scaffolds [115], but comparative studies are needed to see if a sericin scaffold plus systemic nutrition is superior to standard care. The concept of combining nutritional intervention with rehabilitation also deserves rigorous testing — while we assume they are synergistic, trials could quantify how much functional recovery is accelerated by the addition of supplements to an exercise regimen. Lastly, patient-centered outcomes like quality of life, recovery time, or even long-term bone quality are important to document.

## Conclusion

In conclusion, current evidence strongly supports the potential of amino acid supplementation and metabolic modulation to improve geriatric bone healing, but filling these research gaps through well-designed clinical trials and longitudinal studies will be essential. Such research will guide the fine-tuning of dosages, identify any unforeseen risks, and solidify the role of these strategies in standard clinical practice for maxillofacial and orthopedic surgery in the elderly [9]. The coming years should focus on translating this integrative approach from bench to bedside, ultimately aiming to reduce fracture healing times, improve surgical success in osteoporotic patients, and enhance the independence and quality of life of our aging population.

## Data Availability

No datasets were generated or analysed during the current study.

## Abbreviations

ALT: Alanine transaminase
AMP: Adenosine monophosphate
AMPK: AMP-activated protein kinase
ASNS: Asparagine synthetase
ASS: Argininosuccinate synthetase
AST: Aspartate transaminase
ATF4: Activating transcription factor 4
ATP: Adenosine triphosphate
BCAA: Branched-chain amino acids
CBS: Cystathionine *β*-synthase
CGL: Cystathionine *γ*-lyase
CRP: C-reactive protein
CTX: C-terminal telopeptide of type I collagen
eIF2α: Eukaryotic initiation factor 2 alpha
EAA: Essential amino acids
GCN2: General control nonderepressible 2 kinase
GS: Glutamine synthetase
GSH: Glutathione
HIF-1α: Hypoxia-inducible factor 1-alpha
IGF-1: Insulin-like growth factor 1
mTOR: Mechanistic target of rapamycin
mTORC1: mTOR Complex 1
NAC: N-Acetylcysteine
NAD⁺/NADH: Nicotinamide adenine dinucleotide (oxidized/reduced)
NEAA: Nonessential amino acids
NO: Nitric oxide
NMDA: N-methyl-D-aspartate
OAT: Ornithine aminotransferase
P1 NP: Procollagen type I n-terminal propeptide
PHGDH: Phosphoglycerate dehydrogenase
P5 C: *Δ*^1^-Pyrroline-5-carboxylate
P5 CS: Pyrroline-5-carboxylate synthetase
PSAT1: Phosphoserine aminotransferase 1
PSPH: Phosphoserine phosphatase
PYCR: Pyrroline-5-carboxylate reductase
ROS: Reactive oxygen species
TCA: Tricarboxylic acid (cycle)
